# Diagnosis-specific sickness absence as a predictor of mortality: the Whitehall II prospective cohort study

**DOI:** 10.1136/bmj.a1469

**Published:** 2008-10-02

**Authors:** Jenny Head, Jane E Ferrie, Kristina Alexanderson, Hugo Westerlund, Jussi Vahtera, Mika Kivimäki

**Affiliations:** 1 Department of Epidemiology and Public Health, University College London, London; 2 Department of Clinical Neuroscience, Section of Personal Injury Prevention, Karolinska Institutet, Berzelius väg 3, 171 77 Stockholm, Sweden; 3 Stress Research Institute, Stockholm University, SE-106 91, Stockholm; 4 Finnish Institute of Occupational Health, Topeliuksenkatu 41 aA, FIN-00250 Helsinki, Finland

## Abstract

**Objective** To investigate whether knowing the diagnosis for sickness absence improves prediction of mortality.

**Design** Prospective cohort study established in 1985-8. Sickness absence records including diagnoses were obtained from computerised registers.

**Setting** 20 civil service departments in London.

**Participants** 6478 civil servants aged 35-55 years.

**Main outcome measures** All cause, cardiovascular, and cancer mortality until 2004, average follow-up 13 years.

**Results** After adjustment for age, sex, and employment grade, employees who had one or more medically certified spells of sickness absence (>7 days) in a three year period had a mortality 1.7 (95% CI 1.3 to 2.1) times greater than those with no medically certified spells. Inclusion of diagnoses improved the prediction of all cause mortality (P=0.03). The hazard ratio for mortality was 4.7 (2.6 to 8.5) for absences with circulatory disease diagnoses, 2.2 (1.4 to 3.3) for surgical operations, and 1.9 (1.2 to 3.1) for psychiatric diagnoses. Psychiatric absences were also predictive of cancer mortality (2.5 (1.3 to 4.7)). Associations of infectious, respiratory, and injury absences with overall mortality were less marked (hazard ratios from 1.5 to 1.7), and there was no association between musculoskeletal absences and mortality.

**Conclusions** Major diagnoses for medically certified absences were associated with increased mortality, with the exception of musculoskeletal disease. Data on sickness absence diagnoses may provide useful information to identify groups with increased health risk and a need for targeted interventions.

## Introduction

The annual rate of medically certified spells of sickness absence seems to be a good global measure of health.^1^ ^2^ It reflects day to day functioning in occupational settings and predicts mortality at least as well as more established indicators of health such as poor self rated health, minor psychiatric morbidity, and presence of longstanding physical illness, disability, or infirmity.^3^ The reasons for such good predictive power of the overall rate of sickness absence are not well understood. According to one hypothesis, sickness absence spells may be a strong predictor of mortality because they capture a wide range of illnesses that employees experience.^3^ An alternative view is that the increased risk of early mortality is confined to specific reasons for absence such as cancer, heart disease, or surgery for which increased mortality would be expected.

One way to clarify the reasons for the predictive value of sickness absence in relation to mortality is to include diagnoses for sickness absence in the analysis. If sickness absence predicts mortality because it captures the full range of illnesses which separately or in combination increase death risk, then specific diagnoses for absences would not improve prediction of mortality compared with the overall sickness absence rate. In contrast, sickness absence diagnoses would contribute to predictive power if specific diagnoses underlie the association between overall sickness absence and mortality or if the strength of association differs according to diagnosis.

In this study, we investigated whether diagnosis-specific sickness absence predicts subsequent mortality from all causes and from specific causes in the Whitehall II cohort of British civil servants. We also determined whether including information on diagnoses for sickness absence improved the prediction of mortality compared with overall sickness absence rates irrespective of diagnoses.

## Methods

### Participants

The target population for the Whitehall II study was all London based office staff aged 35-55 working in 20 civil service departments. Baseline screening was carried out between 1985 and 1988.^4^ With a response rate of 73%, the final cohort consisted of 10 308 (6895 men, 3413 women). The true response rate was higher, however, because about 4% of those invited were not eligible for inclusion. Ethical approval for the Whitehall II study was obtained from the University College London Medical School committee on the ethics of human research.

### Sickness absence

We obtained computerised sickness absence records from 1 January 1985 onwards from civil service pay centres for 9179 employees. These records included the start and end dates of all absences. For absences longer than seven calendar days, a medical certificate was required.

Diagnosis for absence was recorded in 12 of the 20 participating departments (61% of participants) from 1985 onwards and was recorded for all 20 departments from 1991 onwards. The exposure in this study was medically certified sickness absence during the first three years for which diagnosis was recorded. For the 12 departments with full records of diagnosis for absence, we used data on sickness absence during the three years after the baseline screening date as the exposure; for the other eight departments, where recording of diagnosis was only available from 1991 onwards, we used data on sickness absence during the three years from 1 January 1991 as the exposure time.

Sickness absence diagnoses were coded by the civil service using a detailed coding system based on the international classification of diseases (ICD-8). The coding system was modified in 1990, with codes in the old system (942 codes) mapped on to a smaller number of codes (586) in the new system. We converted the civil service codes to disease categories using the morbidity coding system of the Royal College of General Practitioners (RCGP). The RCGP disease categories are similar to the ICD-8 chapter headings. We modified the RCGP classification by adding four extra disease categories (gastrointestinal, headache and migraine, neurosis, neurosis ill-defined) that accounted for a high proportion of sickness absence spells. Further details on the coding of diagnoses into the 25 disease categories (see box) have been reported previously.^5^

**Modified RCGP disease categories used in this study**

Code 1—Infectious and parasitic diseases
Code 2—Neoplasms
Code 3—Endocrine, nutritional, and metabolic diseases
Code 4—Diseases of blood and blood forming organs
Code 5—Psychoses
Code 6—Diseases of system
Code 7—Diseases of eye
Code 8—Diseases of ear
Code 9—Cardiovascular diseases
Code 10—Cerebrovascular diseases
Code 11—Peripheral vascular diseases
Code 12—Diseases of respiratory system
Code 13—Diseases of digestive system
Code 14—Diseases of genitourinary system
Code 15—Complications of pregnancy, childbirth, and puerperium
Code 16—Diseases of skin and subcutaneous tissue
Code 17—Diseases of musculoskeletal system and connective tissue
Code 20—Symptoms, signs, and ill-defined conditions
Code 23—Injury and poisoning
Code 35—General medical examinations and screening
Code 37—Surgical operations
Code 38—Gastrointestinal
Code 39—Headache and migraine
Code 40—Neurosis
Code 41—Neurosis ill-defined

We assessed the validity of the civil service diagnosis codes by obtaining information from participants’ general practitioners for all absence spells lasting >21 days in 1985-90: the civil service codes and general practitioners showed agreement on disease category for 64% of these absences.^6^ We also checked the sickness absence records for inconsistencies: we merged overlapping, consecutive, or duplicate spells of sickness absence after taking account of weekends and public holidays, and used the final diagnosis for absence when we merged contiguous spells.

### Mortality

We obtained mortality data for almost all the participants (n=10 297) up to 30 September 2004 from the National Health Service Central Register mortality register. In addition to deaths from all causes, we analysed deaths from cardiovascular diseases (ICD-9 codes 390-459, ICD-10 codes I00-I99) and cancer (ICD-9 140-208, ICD-10 C00-C97). Smoking related cancers were considered to be ICD-9 codes 140, 141, 143-150, 157, 160-163, 188, and 189 and ICD-10 codes C00-C06, C09-C15, C25, C30-C34, C38, and C64-C68.^7^

### Other measures

Smoking, alcohol consumption (units consumed in previous 7 days), presence of hypertension (use of antihypertensive drugs or blood pressure >160/95 mm Hg), body mass index (weight (kg)/(height (m)^2^), self rated health over the past 12 months, and presence of longstanding illness, disability, or infirmity were recorded at baseline. We created a composite physical illness indicator (diabetes, diagnosed heart trouble, electrocardiographic abnormalities, hypertension, and respiratory illness). Self reported diagnosis of cancer was recorded at baseline and at phase 4 follow-up of the Whitehall II study (1995-6).There were missing data for the measure of longstanding illness as this was introduced only after the start of the baseline survey: where data were missing, we used reported longstanding illness from the phase 2 follow-up survey (1989-90).

### Statistical analysis

For each employee, we computed the numbers of medically certified spells (>7 days) of sickness absence for each diagnostic category during the three year exposure period. Diagnostic categories with small numbers of participants were combined into an “other” category. Follow-up for mortality was from the first day after the three year exposure period until 30 September 2004. We used Cox’s proportional hazard models to estimate hazard ratios for having taken one or more spells of absence for each sickness absence diagnostic category. The reference group was participants who had no sickness absence spells lasting >7 days during the exposure period. All analyses were adjusted for sex, age (in 5 year groups), and employment grade. If participants had two (or more) spells of sickness absence in different diagnostic categories, they were included in both categories.

Using the likelihood ratio χ^2^ test, we tested for an improvement in predictive power for mortality by taking account of absence diagnoses. We compared a multivariate model including indicators for the main diagnostic categories with the simpler model ignoring diagnoses (any medically certified sickness absences versus no such absences). We tested for the possibility of differences in associations for women and men by including an interaction term with sex. Further analyses adjusted for other known predictors of mortality (smoking, alcohol consumption, hypertension, and body mass index). We tested the proportional hazards assumption by including an interaction of each diagnosis-specific absence variable with the log of follow-up time; in all cases, this interaction was not statistically significant. We used the SAS 9.1 program for all analyses.

## Results

### Excluded participants

Of the 10 308 participants in the Whitehall II study, 3830 were excluded from our analysis (figure[Fig fig1]). Reasons for exclusion were missing data on mortality (n=11) or absence records (n=1124) and an incomplete three year exposure period (n=2695; 120 died and 2575 left the civil service during the exposure period). We compared mortality after 1993 in the remaining sample of 6478 participants with the sample excluded from analysis. After adjustment for age, sex, and employment grade, mortality was lower in the analysed sample than in the excluded sample (hazard ratio 0.81, 95% confidence interval 0.67 to 0.98).

**Figure fig1:**
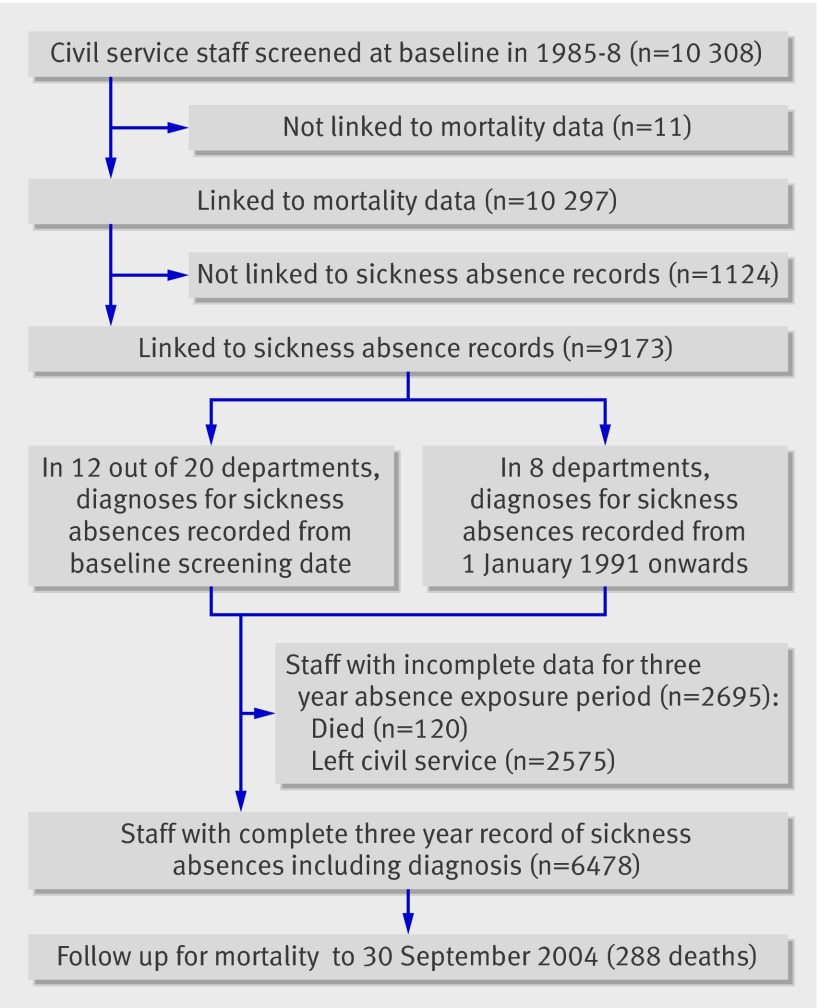
Flow of participants through study

### Overall sickness absence and mortality

Of the 6478 participants, 288 died during the follow-up period from the first day after the sickness absence exposure to September 2004. Mean follow-up was 13.2 (SD 2.3, range 0.1-16.1) years. Mortality in the 12 civil service departments where absence diagnoses were recorded from baseline was similar to that in the other eight departments where recording began in 1991 (hazard ratio 0.98, 0.74 to 1.31).

Table 1 shows the numbers of participants who had one or more spells of medically certified sickness absence during the three year exposure period. A total of 1906 employees (29%) had one or more such spells, 18% having one spell only and 11% having two or more. Altogether, there were 3214 such spells of absence, with a median length of 16 days.

**Table 1 tbl1:** Distribution of spells of medically certified sickness absence during three year exposure period among civil service employees

	No (%) of participants (n=6478)	No (%) of spells of sickness absence
Any medically certified spells of sickness absence (lasting >7 days)	1906 (29.4)	3214 (100)
No of medically certified spells in three years:		
0	4572 (70.6)	—
1	1170 (18.1)	—
2	415 (6.4)	—
3	183 (2.8)	—
>3	138 (2.1)	—
Diagnostic category for medically certified spells (category code)*:		
Infectious (1)	244 (3.8)	274 (8.5)
Respiratory (12)	685 (10.6)	850 (26.4)
Musculoskeletal (17)	257 (4.0)	305 (9.5)
Circulatory (9, 10, 11)	61 (0.9)	71 (2.2)
Injury (23)	230 (3.6)	255 (7.9)
Psychiatric (40, 41)	235 (3.6)	303 (9.4)
Surgery (37)	318 (4.9)	364 (11.3)
Other (2-8, 13-16, 20, 35, 38, 39)	377 (5.8)	461 (14.3)
Not codeable or missing	289 (4.5)	331 (10.3)
Multiple medically certified spells†:		
2 spells same diagnosis	105 (1.7)	—
2 spells different diagnoses	231 (3.7)	—
>2 spells same diagnosis	27 (0.4)	—
>2 spells different diagnoses	214 (3.5)	—

*Modified RCGP morbidity coding system (see text for details).

†Among the 6189 participants with no missing or uncodeable diagnoses.

Table 2 shows the associations of these spells of absence with subsequent mortality, (adjusted for age, sex, and employment grade). Among the participants who had had one or more medically certified absence in the three year exposure period mortality was 1.7 times higher than among those with no such absences, and further adjustment for self rated health, longstanding illness, and the composite physical illness indicator had little effect on this (hazard ratio 1.59, 1.16 to 2.17). We also found a dose-response association between rates of medically certified absence with subsequent mortality: compared with having no medically certified absences, the hazard ratio for having two or more medically certified absences was 1.97 (1.43 to 2.71) and for having one such absence was 1.48 (1.11 to 1.98).

**Table 2 tbl2:** Hazard ratios for all cause mortality (adjusted for age, sex, and employment grade) among 6478 civil service employees by specific diagnoses for medically certified spells of sickness absence

Diagnostic category (category code)*	No of participants (deaths)	Hazard ratio (95% CI)
No medically certified sickness absence	4570 (161)	1.00
Any medically certified absence (all diagnoses):	1906 (127)	1.66 (1.30 to 2.13)
Infectious and parasitic diseases (1)	244 (15)	1.51 (0.88 to 2.59)
Circulatory (9-11)	61 (12)	4.68 (2.58 to 8.51)
Diseases of respiratory system (12)	685 (46)	1.63 (1.15 to 2.29)
Diseases of musculoskeletal system and connective tissue (17)	257 (12)	1.04 (0.57 to 1.89)
Injury and poisoning (23)	230 (17)	1.66 (0.99 to 2.78)
Surgical operations (37)	318 (27)	2.16 (1.42 to 3.26)
Psychiatric (40, 41):	235 (19)	1.91 (1.17 to 3.11)
Neurosis (40)	87 (7)	1.91 (0.89 to 4.11)
Neurosis ill-defined (41)	164 (14)	2.03 (1.16 to 3.55)
Other (2-8, 13-16, 20, 35, 38, 39)	377 (27)	1.78 (1.17 to 2.70)
Diagnosis not codeable or missing	289 (22)	2.09 (1.33 to 3.30)

*Modified RCGP morbidity coding system (see text for details).

### Diagnosis-specific sickness absence and mortality

The commonest diagnostic categories were respiratory, surgery, musculoskeletal, psychiatric, infectious diseases, and injury—which together accounted for 73% of all medically certified absences in the three year exposure period. Among those participants who had two or more absences, 77% had absences attributed to two or more of the 25 different diagnostic categories.

As shown in table 2, medically certified absences attributed to infectious diseases, respiratory diseases, and injury had similar hazard ratios for mortality, ranging from 1.5 to 1.7. The increased mortality risk was greater, but not significantly so, for spells of absence with a non-psychotic psychiatric diagnosis (hazard ratio 1.9), and this increased risk was seen with both subcategories “neurosis” (1.9) and “neurosis ill-defined” (2.0). Hazard ratios were higher for sickness absences attributed to circulatory disease (4.7) and surgical operations (2.2). Fewer than 10 people had sickness absence attributed to cancer diagnoses; this group had a particularly high hazard ratio of 21.3 (6.7 to 67.4). Those having absence with a musculoskeletal diagnosis did not have increased mortality compared with participants who took no medically certified absences (hazard ratio 1.0).

Comparison of our models with and without sickness absence diagnoses indicated that inclusion of diagnoses significantly improved the prediction of mortality (P for improvement in χ^2^=0.03). This statistical test of improvement in model fit is equivalent to testing whether hazard ratios for mortality vary by diagnosis. The only diagnosis where the hazard ratio for all cause mortality significantly exceeded the hazard ratio of 1.66 for all absences was circulatory disease (P<0.001).

None of the interactions between diagnostic category and sex was significant. Having multiple absences with two or more different diagnoses during the three year exposure period was associated with slightly higher mortality, but this was not significant (hazard ratio 1.34, 0.75 to 2.39, for ≥2 absences with different diagnoses *v* ≥2 absences with same diagnosis).

We repeated analyses after excluding the 31 employees who died in the three years immediately after the three year sickness absence exposure period, to exclude the possibility that absence close to death might account for our findings. The hazard ratio for any medically certified absence compared with no such absence was 1.59 (1.23 to 2.07). Likewise, hazard ratios for diagnosis-specific absence were also hardly altered (results not shown).

### Sickness absence and cause-specific mortality

Table 3 presents associations between medically certified spells of absence and cause-specific mortality. The two leading causes of death were cancer (144 deaths) and cardiovascular mortality (72 deaths), accounting for 50% and 25% of all deaths. Other causes (68 deaths) accounted for 24% of all deaths. Cause was unknown for four deaths. Among the cancer deaths, 30% were assessed as smoking related.

**Table 3 tbl3:** Hazard ratios for cause-specific mortality (adjusted for age, sex, and employment grade) among 6478 civil service employees by specific diagnoses for medically certified spells of sickness absence

Diagnostic category (category code)*	Mortality							
	Cardiovascular (72 deaths)			Neoplasms (144 deaths)			Other (68 deaths)	
	No of deaths	Hazard ratio (95% CI)		No of deaths	Hazard ratio (95% CI)		No of deaths	Hazard ratio (95% CI)
No medically certified sickness absence	36	1.00		80	1.00		41	1.00
Any medically certified absence (all diagnoses):	36	1.96 (1.20 to 3.21)		64	1.73 (1.22 to 2.45)		27	1.39 (0.83 to 2.33)
Infectious and parasitic diseases (1)	5	2.14 (0.82 to 5.60)		6	1.24 (0.53 to 2.88)		4	1.56 (0.55 to 4.46)
Circulatory (9-11)	5	6.20 (2.35 to 16.3)		3	2.59 (0.81 to 8.29)		4	6.51 (2.27 to 18.7)
Diseases of respiratory system (12)	15	2.23 (1.18 to 4.21)		21	1.52 (0.92 to 2.51)		10	1.40 (0.68 to 2.88)
Diseases of musculoskeletal system and connective tissue (17)	3	0.98 (0.30 to 3.25)		5	0.93 (0.37 to 2.33)		4	1.40 (0.49 to 4.00)
Injury and poisoning (23)	4	1.62 (0.50 to 4.67)		4	0.82 (0.30 to 2.28)		9	3.58 (1.68 to 7.66)
Surgical operations (37)	8	2.85 (1.31 to 6.22)		12	1.94 (1.05 to 3.59)		7	2.19 (0.97 to 4.95)
Psychiatric (40, 41)	3	1.24 (0.37 to 4.09)		12	2.49 (1.33 to 4.68)		4	1.61 (0.56 to 4.62)
Other (2-8, 13-16, 20, 35, 38, 39)	8	2.16 (0.98 to 4.75)		12	1.63 (0.87 to 3.02)		7	1.83 (0.80 to 4.17)
Diagnosis not codeable or missing	4	1.64 (0.57 to 4.69)		14	2.77 (1.54 to 4.97)		4	1.46 (0.51 to 4.16)

*Modified RCGP morbidity coding system (see text for details).

Medically certified spells of sickness absence were associated with both cardiovascular mortality (hazard ratio 2.0) and cancer mortality (hazard ratio 1.7). Absence attributable to psychiatric disorder showed a stronger association with cancer mortality (hazard ratio 2.5) than with cardiovascular mortality (hazard ratio 1.2), but the difference was not significant. The association with cancer mortality was seen for both psychiatric categories—“neurosis” (hazard ratio 2.2, 0.8 to 6.2) and “neurosis ill-defined” (3.0, 1.5 to 5.9)—and was also observed in the subgroup whose absences were all attributable to psychiatric disorder (hazard ratio 2.4). To exclude the possibility that this association might be explained by higher rates of sickness absence for psychiatric causes among people with cancer diagnosed, we repeated the analysis excluding the 166 participants with a self reported diagnosis of cancer either at baseline or follow-up in 1995-6. In the subgroup without cancer, participants who had absences with a psychiatric diagnosis had cancer mortality 2.4 times higher (95% CI 1.2 to 4.6) than did those with no absence.

Otherwise, rates of diagnosis-specific absence tended to show stronger associations with cardiovascular mortality than cancer mortality, although not significantly so. However, absences with a musculoskeletal diagnosis were not associated with either cardiovascular or cancer mortality. None of the interactions between sex and diagnosis-specific absence were significant either for cancer mortality or cardiovascular mortality. Medically certified absences were associated with mortality from suicide (ICD-9 code E95, ICD-10 codes X60-X84) or external cause of undetermined intent (ICD-9 E98, ICD-10 Y10-Y34) (six deaths, hazard ratio 6.9, 1.2 to 39.1).

Adjustment for smoking, alcohol consumption, body mass index, and hypertension did not remove the associations of medically certified sickness absence with cause-specific mortality (table 4). As might be expected, the association between absences with a circulatory diagnosis and cardiovascular mortality was somewhat reduced after adjustment for these risk factors, but it remained significant (hazard ratio 3.4). However, adjustment for these risk factors only slightly attenuated the increased risk of cardiovascular mortality associated with absences for other diagnoses. Absences for both psychiatric and non-psychiatric causes maintained their associations with cancer mortality after adjustment for risk factors.

**Table 4 tbl4:** Hazard ratios for cause-specific mortality among civil service employees by specific diagnoses for medically certified spells of sickness absence

Diagnostic category*	Hazard ratio (95% CI)		
	Adjusted for age, sex, and grade (model A)	Model A + adjusted for smoking, alcohol, body mass index, and hypertension	Model A with three year washout period†
**Cardiovascular mortality**	**(68 deaths/6255 participants)**		**(59 deaths/6218 participants)**
No medically certified sickness absence	1.00	1.00	1.00
Any medically certified absence (all diagnoses):	2.24 (1.35 to 3.71)	1.97 (1.18 to 3.30)	2.18 (1.28 to 3.36)
Circulatory	5.93 (2.04 to 17.3)	3.42 (1.13 to 10.4)	7.71 (2.62 to 22.7)
Other diagnoses	2.08 (1.24 to 3.49)	1.88 (1.11 to 3.19)	1.97 (1.12 to 3.46)
**Cancer mortality**	**(134 deaths/6255 participants)**		**(118 deaths/6218 participants)**
No medically certified sickness absence	1.00	1.00	1.00
Any medically certified absence (all diagnoses):	1.84 (1.28 to 2.65)	1.75 (1.21 to 2.52)	1.83 (1.24 to 2.70)
Psychiatric	2.64 (1.37 to 5.08)	2.44 (1.26 to 4.70)	2.27 (1.07 to 4.82)
Other diagnoses	1.74 (1.19 to 2.54)	1.65 (1.13 to 2.43)	1.77 (1.18 to 2.65)

*Modified RCGP morbidity coding system (see text for details).

†Washout period=exclusion of participants who died in the first three years after assessment of sickness absence.

Exclusion of participants who died in the first three years after assessment of sickness absence had little effect on hazard ratios for cause-specific mortality (table 4). Similarly, when deaths in the first five years were excluded, there was still a twofold increase in cancer mortality among those having absences for psychiatric reasons.

## Discussion

This study shows that knowing the diagnosis for medically certified sickness absence from work significantly improves the prediction of mortality. Employees taking one or more medically certified spells of absence (>7 days) for the common diagnostic categories had increased mortality compared with colleagues taking no medically certified absence. The only exception was musculoskeletal absence, which was not associated with increased mortality. Unexpectedly, employees who had one or more absence for psychiatric reasons had a considerable 2.5-fold greater cancer mortality.

### Comparison with other studies

Previous studies have shown that medically certified absence rates predict all cause mortality,^3^ ^8^ but to our knowledge only one study, of very long term sickness absence (>8 weeks),^9^ has linked diagnosis-specific sickness absence with mortality. While we found associations with mortality for most diagnosis-specific absences, this association was particularly strong for absences with a cardiovascular diagnosis and for absences with a psychiatric diagnosis, which is one of the most common diagnoses. We found no association between rates of absence for a musculoskeletal reason and mortality—which is as expected as musculoskeletal disorders rarely cause death. The increased risk of mortality among the few people who had absences because of cancer is in line with findings from the above mentioned study of long term sickness absence in Norway.^9^

Few studies on sickness absence have examined cause-specific mortality. We found that sickness absence predicts both cardiovascular and cancer mortality, the two leading causes of death in Western societies, replicating findings for Finnish municipal employees.^8^ In that Finnish study the association between sickness absence and cancer was stronger for cancers not attributable to smoking. In our study most of the cancer deaths (70%) were not considered to be smoking related,^7^ and adjustment for smoking, alcohol consumption, and body mass index made little difference to our results, suggesting that the association between sickness absence and cancer mortality is not due to these risk factors.

Why is sickness absence with a psychiatric diagnosis related to all cause and cancer mortality? This was not due to confounding by health behaviours because adjustment for smoking and alcohol consumption hardly altered our results. Several studies have found that depression and negative affective dispositions, such as neuroticism and dispositional anxiety, are risk factors for all cause mortality and cardiovascular mortality.^10^ ^11^ ^12^ ^13^ The large population based Norwegian HUNT study showed that depression predicted subsequent cancer mortality as well as cardiovascular mortality.^14^ One possible explanation for the association is that depression may interfere with help seeking behaviour,^14^ as survival is often related to early detection of cancer. A further possible explanation is that psychiatric disorders affect cancer prognosis rather than aetiology.^15^ Depression may also impair adherence to cancer treatment, although recent reviews on breast cancer found no conclusive evidence that psychological factors influence survival or relapses.^16^ ^17^ It is also possible that these psychiatric diagnoses are early symptoms of undiagnosed cancer. For example, the subcategory “neurosis ill-defined” included tiredness and stress, which could be early symptoms of undiagnosed cancer or other physical illness. However, excluding deaths in the first three years did not alter our results, and we still found a twofold increase in cancer mortality after we excluded deaths in the first five years.

### Strengths and limitations

The strengths of our study were the large sample covering a wide range of employment grades from low to high incomes, the long follow-up for mortality, the use of reliable mortality registers, and the high validity of sickness absence data that included information on diagnosis. 

Some limitations are noteworthy. Firstly, some data on sickness absence diagnosis were missing in the early years of the Whitehall II study. This is unlikely to have led to biased results, as associations of overall sickness absence with mortality from all causes, cancer, and cardiovascular disorders were similar in the total sample and in the sample analysed in this study. 

Secondly, numbers of deaths were small in some of the diagnostic categories for sickness absence, and our results therefore need replication. There was insufficient power for detailed analyses of, for example, duration of sickness absence spells or less common diagnoses for absence. Our study also had low power to detect sex differences or to examine whether the association of sickness absence with mortality differed according to employment grade. Previous findings from this cohort showed that there are sex differences in rates of medically certified absences after taking account of age and employment grade: women had higher rates for absences with respiratory, psychiatric, or surgery diagnoses; similar rates for musculoskeletal and injury; and a non-significantly lower rate for cardiovascular diagnoses.^6^

Thirdly, the recorded diagnosis for a sickness absence may not cover all of the actual causes as sickness absence is a complex multifactorial behaviour and there may not be a simple relation between illness and taking of sick leave.^18^ Only one diagnosis is recorded for each sick leave, and thus coexisting diseases are not covered. Moreover, the stage and severity of disease when affected individuals decide to take sick leave may vary between diagnoses, and this might account for some of the differences in excess mortality between sickness absence diagnoses.

### Conclusions and policy implications

Sickness absence is a serious economic problem with large costs for health care and many lost working hours for businesses.^2^ ^18^ We found that the almost 30% of participants who had a sickness absence spell of more than seven days over a three year period had a 66% increased risk of premature death. We do not consider that the taking of sick leave itself is a risk behaviour. Instead, it may be a marker of circumstances and health problems that increase mortality. We believe that better monitoring of diagnosis-specific absences at population levels may be useful and contribute to policy making—not only as a measure of costs but also as data to identify groups at increased health risk and in need of targeted intervention.

**What is already known on this topic**

Rates of medically certified sickness absence from work are associated with increased all cause mortality
It is not known whether this association is restricted to specific diagnoses for sickness absence

**What this study adds**

All the common diagnostic categories for sickness absence predicted overall mortality, with the exception of musculoskeletal diagnoses
Sickness absence with a psychiatric diagnosis, one of the commonest diagnoses for medically certified absence, was a strong predictor of cancer related mortality
Routinely collected data on sickness absences that include the diagnosis may help to identify groups at increased risk of fatal disease and allow targeted early interventions

## Notes

We thank all participating civil service departments and their welfare, personnel, and establishment officers; the Occupational Health and Safety Agency; the Council of Civil Service Unions; all participating civil servants in the Whitehall II study; all members of the Whitehall II study team.

Ethical approval: Ethical approval for the Whitehall II study was obtained from the University College London Medical School committee on the ethics of human research.
